# A Transcriptome Analysis Reveals that Hepatic Glycolysis and Lipid Synthesis Are Negatively Associated with Feed Efficiency in DLY Pigs

**DOI:** 10.1038/s41598-020-66988-6

**Published:** 2020-06-18

**Authors:** Cineng Xu, Xingwang Wang, Zhanwei Zhuang, Jie Wu, Shenping Zhou, Jianping Quan, Rongrong Ding, Yong Ye, Longlong Peng, Zhenfang Wu, Enqin Zheng, Jie Yang

**Affiliations:** College of Animal Science and National Engineering Research Center for Breeding Swine Industry, South China Agricultural University, Guangdong, P.R. China

**Keywords:** Gene expression, RNA sequencing, Transcriptomics

## Abstract

Feed efficiency (FE) is an important trait in the porcine industry. Therefore, understanding the molecular mechanisms of FE is vital for the improvement of this trait. In this study, 6 extreme high-FE and 6 low-FE pigs were selected from 225 Duroc × (Landrace × Yorkshire) (DLY) pigs for transcriptomic analysis. RNA-seq analysis was performed to determine differentially expressed genes (DEGs) in the liver tissues of the 12 individuals, and 507 DEGs were identified between high-FE pigs (HE- group) and low-FE pigs (LE- group). A gene ontology (GO) enrichment and pathway enrichment analysis were performed and revealed that glycolytic metabolism and lipid synthesis-related pathways were significantly enriched within DEGs; all of these DEGs were downregulated in the HE- group. Moreover, Weighted gene co-expression analysis (WGCNA) revealed that oxidative phosphorylation, thermogenesis, and energy metabolism-related pathways were negatively related to HE- group, which might result in lower energy consumption in higher efficiency pigs. These results implied that the higher FE in the HE- group may be attributed to a lower glycolytic, energy consumption and lipid synthesizing potential in the liver. Furthermore, our findings suggested that the inhibition of lipid synthesis and glucose metabolic activity in the liver may be strategies for improving the FE of DLY pigs.

## Introduction

Feed efficiency (FE) is an important economic trait in the porcine industry, as feed cost accounts for approximately 70% of the total production cost^1^. Therefore, improving FE can greatly promote the economic benefits of pig production. The main indicators for measuring FE are residual feed intake (RFI) or feed conversion ratio (FCR). RFI is defined as the difference between the animal’s actual feed intake and its predicted dry matter intake (DMI) based on production needs, to specifically capture the efficiency of feed use independent from production needs^2^. A lower RFI value indicates a more efficient pig. FCR is the ratio of feed intake to the average daily gain (ADG) during a specified period. Compared with FCR, RFI can more accurately reflect the differences of FE in pigs with different body weights and growth rate^2^. Therefore, RFI is preferred as the selection indicator. Because of the strong genetic correlation with RFI (R equals 0.76–0.99)^3^, FCR can be used as a reference indicator to verify the selection based on RFI, which can measure FE more intuitively.

To date, based on genome-wide association analysis (GWAS) of pigs, some SNPs and candidate genes that might affect FE have been identified. SNPs located on SSC7, SSC13, SSC14, and SSC17 and candidate genes, such as *MBD5*, *GTF3C5*, *HMGA2*, *PITX2*, and *MAP3K14*, were reported to be associated with FE in the previous studies^4–7^. However, GWAS studies are limited to finding chromosomal regions or preselected genes that affect FE^8^ and finding candidate genes and pathways that affect FE is difficult. Instead, RNA-seq technology can quantitatively measure gene expression in individuals to screen differentially expressed genes (DEGs)^9,10^. By analyzing the DEGs and related biological pathways, candidate genes and pathways that affect FE can be identified.

RNA-seq has been used to research FE in animals, including pigs, cattle, and poultry, and muscle, liver, and adipose tissues have been used as research materials^11–13^. Recently, a growing number of transcriptome analysis has focused on the liver tissue of pigs to identify candidate genes and pathways associated with FE^14–16^. In mammals, the liver plays a prominent and central role in regulating the metabolism and distribution of nutrients. On the one hand, macronutrients, such as carbohydrates, lipids, and proteins, are metabolized in the liver^17,18^. On the other hand, the liver can synthesize and store nutrients, as well as release nutrients into the blood^19^. Several studies have revealed that lipid metabolism, such as fatty acid synthesis, lipogenesis, and steroidogenesis, in the liver tissue was altered in FE-divergent pigs^14,20,21^. In addition, glucose metabolism and energy metabolism in the liver have been reported to be essential for the regulation of FE in pigs, and lower glycolytic potential and energy loss were found in high-FE pigs^15^.

Many transcriptome studies focused on purebred pigs (including Yorkshire and Duroc) and crossbred pigs, such as Large White × (Landrace × Pietrain) and Maxgro × (Landrace × Large White) pigs, have made some progress in identifying candidate genes and pathways linked with FE^14,20–22^. However, none of the transcriptome studies have been conducted in the liver of commercial Duroc × (Landrace × Yorkshire) (DLY) pigs, while DLY pigs are by far the largest population in the porcine industry worldwide^23^. As a result, in this study, we used RNA-seq technology to profile the liver transcriptome of 6 extremely high-FE DLY pigs (HE- group) and 6 extremely low-FE DLY pigs (LE- group) to identify candidate genes and pathways that significantly correlated with the FE of DLY pigs. Furthermore, the identified genes and pathways that affect FE can provide theoretical support for pig selection, to improve the feed efficiency and economic benefits in commercial pig production in the future.

## Results

### Phenotypic parameters in Pigs from the HE- and LE- groups

**Figure 1:**
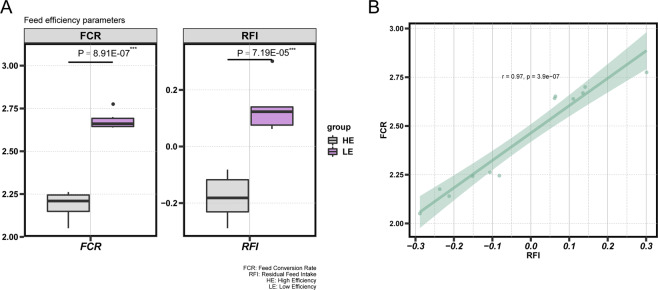
Feed efficiency (FE) phenotypic parameters in Duroc × (Landrace × Yorkshire) (DLY) pigs from high-FE pigs (HE- group) and low-FE pigs (LE- group). (**A**) Boxplot of the feed conversion ratio (FCR) and residual feed intake (RFI) in the two groups. (**B**) The correlation coefficient between FCR and RFI in the two groups.

Six extremely high-FE pigs had the phenotypic parameter of RFI = −0.18 ± 0.08 and FCR = 2.19 ± 0.08, while six extremely low-FE pigs had the phenotypic parameter of RFI = 0.14 ± 0.09 and FCR = 2.68 ± 0.05. The phenotypic details of the HE- and LE- groups are shown in Table S1. The FCR and RFI of the HE- group were significantly lower than those of the LE- group, which are displayed in the boxplot (Fig. 1A); thus, the HE- group was more efficient than the LE- group. A high linear positive correlation between FCR and RFI (Fig. 1B) was identified in our study, which was consistent with previous studies^3^.

### Mapping statistics summary

In this study, the mapping statistics summaries for each sample are listed in Table S2. On average, the sequencing generated 40719764 and 40985844 effective reads in the HE- and LE- groups, respectively. Among the effective reads, on average, 93.36% and 92.81% in the HE- and LE- groups, respectively, were uniquely mapped to the reference genome, and 3.62% and 3.69% were multiple mapped to the reference genome.

### A total of 507 DEGs between the HE- and LE- groups

**Figure 2:**
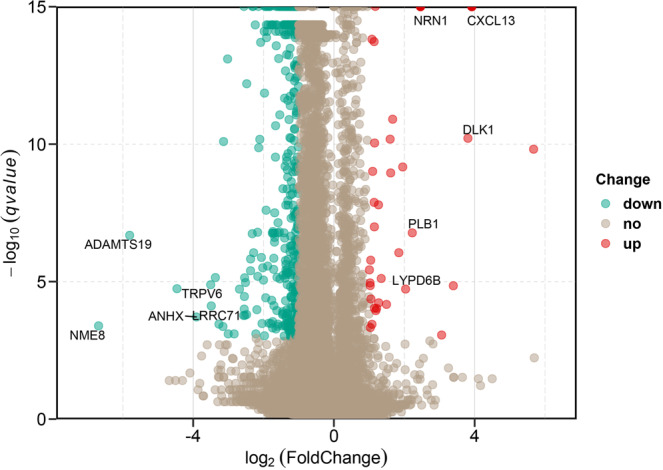
Differentially expressed genes (DEGs) between the HE- and LE- groups. A plot of DEGs with |log_2_(Foldchange)| > 1 and *q*-value < 0.001. Red dots represent significantly upregulated genes, and green dots represent significantly downregulated genes. The genes marked in the figure are the 5 most significantly upregulated and 5 most downregulated named genes. The x-axis and y-axis represent the −log_10_(qvalue) and log_2_(Foldchange), respectively.

In the current study, a total of 507 DEGs satisfied the criteria of |log_2_(Foldchange)| > 1 and *q*-value < 0.001. Among the 507 DEGs, 53 DEGs were upregulated and 454 DEGs were downregulated in the HE- group; the 5 most significantly upregulated named genes (including *NRN1, CXCL13, DLK1, PLB1, and LYPD6B*) and 5 most significantly downregulated named genes (including *ADAMTS19, TRPV6, NME8, ANHX, and LRRC71*) are marked (Fig. 2). The details of all DEGs with their log_2_(Foldchange) and *q*-value are listed in Table S3.

### GO enrichment analysis of DEGs

The biological process (BP), molecular function (MF), and cellular component (CC) GO terms of 507 DEGs were identified and the details of all identified GO terms are listed in Table S4. A total of 5 GO terms were significantly enriched (*q-*value <0.05), including 1 BP_GO term and 4 MF_GO terms. All of the genes enriched in the 5 GO terms were downregulated in the HE- group (Table 1). None of the CC_GO terms was significantly enriched. The significantly enriched BP_GO term was carbohydrate phosphorylation, which is involved in glycolysis. The most significantly enriched MF_GO term was carbohydrate kinase activity. Carbohydrate kinase includes hexokinase, phosphofructokinase, and other kinases, which mainly catalyze glycolysis. Genes involved in the 2 terms were downregulated in the HE- group, indicating that glycolysis might decrease in the liver of the HE- group. The remaining 3 MF_GO terms were related to guanyl-nucleotide exchange factor activity, which exchanges GDP for GTP^24^, and the DEGs enriched in these terms were downregulated in the HE- group.

**Table 1:** Significantly enriched GO terms. Enriched liver DEGs between the HE- and LE- groups according to gene ontology (GO) terms for biological processes, molecular function, and cellular component.

GO_ID	GO term	*q-*value	Gene Names
**Biological process**			
GO:0046835	carbohydrate phosphorylation	4.99E-03	*HK3, PFKFB4, PFKFB1, GCK*
**Molecular function**			
GO:0019200	carbohydrate kinase activity	1.01E-03	*HK3, PFKFB4, PFKFB1, GCK*
GO:0005088	Ras guanyl-nucleotide exchange factor activity	1.68E-03	*ARHGEF37, ARHGEF39, ARHGEF16, ARHGEF25, DENND3*
GO:0005089	Rho guanyl-nucleotide exchange factor activity	2.89E-03	*ARHGEF39, ARHGEF16, ARHGEF37, ARHGEF25*
GO:0005085	guanyl-nucleotide exchange factor activity	7.78E-03	*ARHGEF37, ARHGEF39, ARHGEF16, ARHGEF25, DENND3*

Significantly enriched terms (*q-*value < 0.05) are listed with the GO_ID, term, *q-*value and gene name. The remaining terms are shown in Table S4.

### Pathway enrichment analysis of DEGs

Our results showed that 25 significantly enriched pathways (*q-*value <0.05) were enriched in the Reactome or KEGG database, 24 pathways were enriched in the Reactome database, and one pathway was enriched in the KEGG database (Table S5). The top 10 significantly enriched pathways and the genes contained in each pathway are listed in Table 2. Among the 10 pathways, 6 pathways were related to carbohydrate metabolism, including metabolism of carbohydrates, glucose transport, glycolysis, hexose transport, glucose metabolism, and starch and sucrose metabolism; the other 4 pathways were correlated with lipid synthesis, including lipid and lipoprotein metabolism, SREBP activation gene expression, SREBP regulation of cholesterol biosynthesis and phase 1 - functionalization of compounds. Most of the genes involved in the 10 pathways were downregulated in the HE- group. These results indicated that decreased lipid and glucose metabolism activity might occur in the liver of the HE- group.

**Table 2:** Top 10 significantly enriched pathways enriched in the Reactome and KEGG databases.

Pathway	*q-*value	Gene Names
**Reactome**		
Metabolism of carbohydrates	5.52E-07	*GCK, HK3, PFKFB1, PFKFB4, LOC100521789, RAE1, FGF21, LOC100739542, LOC100522672, CHST1*
Glucose transport	4.82E-05	*GCK, HK3, FGF21, RAE1, LOC100739542*
Glycolysis	4.82E-05	*GCK, HK3, PFKFB1, PFKFB4*
Hexose transport	4.82E-05	*GCK, HK3, FGF21, RAE1, LOC100739542*
Glucose metabolism	0.001906	*GCK, HK3, PFKFB1, PFKFB4*
Metabolism of lipids and lipoproteins	0.0029006	*ACACB, SCD, CYP21A2, CHKA, CYP7A1, HSD17B1, PLA2G4B, LSS, HMGCS2*
Activation of gene expression by SREBP	0.0098664	*ACACB, SCD, LSS*
Regulation of cholesterol biosynthesis by SREBP	0.0099889	*ACACB, SCD, LSS*
Phase 1 - Functionalization of compounds	0.0124725	*CYP21A2;AOC1;CYP7A1*
**KEGG pathway**		
Starch and sucrose metabolism	0.039301	*GCK, HK3, UGT1A1, LOC100516628, LOC100521789, GBE1, LOC100522672*

Enriched DEGs according to the Reactome or Kyoto Encyclopedia of Genes and Genomes (KEGG) database are shown. Significantly enriched pathways (*q*-value < 0.05) are listed with the pathway, *q*-value and gene names. Twenty-four pathways were significantly enriched in the Reactome database, while only 1 pathway was significantly enriched in the KEGG database. The top 10 significantly enriched pathways are listed. The remaining pathways are shown in Table S5.

### Protein-protein interaction (PPI) analysis

**Figure 3:**
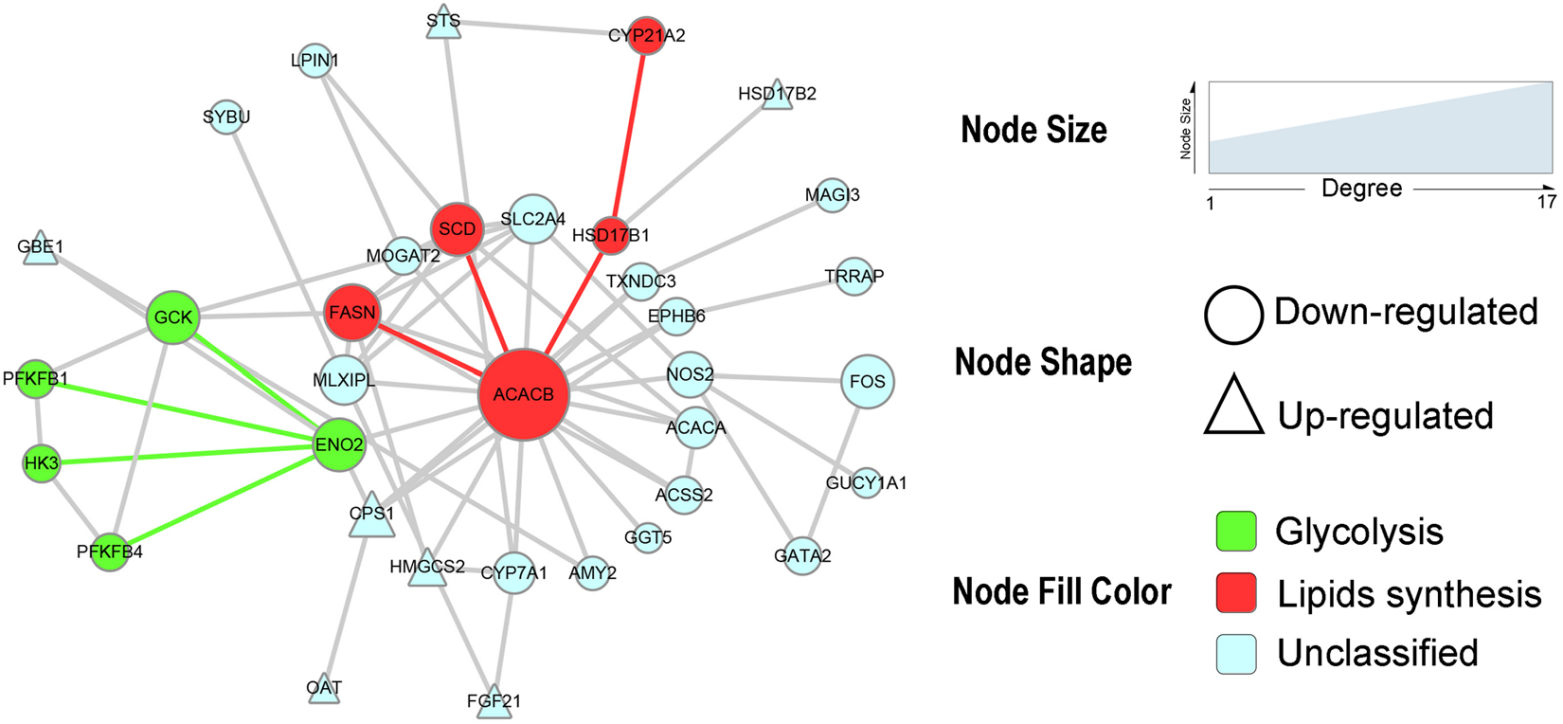
The key network of DEGs in the liver from HE- compared with LE- group. The network diagram centers on the *ACACB* gene, which has the largest degree of change, and the DEGs are directly or indirectly related to *ACACB*. Node shape represents the change in gene expression. The node fill color represents the functional classification of the gene.

According to Protein-protein interaction (PPI) analysis, a network of some of the named genes was visualized to explore the interaction of them with each other (Fig. 3). The network diagram was centered on the *ACACB* gene, which had the largest degree, and 32 DEGs were directly or indirectly related to it. Genes highlighted in green (*GCK*, *HK3*, *ENO2*, *PFKFB1*, and *PFKFB4*) are involved in glycolysis, and genes highlighted in red (*ACACB*, *SCD*, *FASN*, *HSD17B1*, and *CYP21A2*) are involved in lipid synthesis. All genes highlighted in green or red color were downregulated in the HE- group, indicating that glycolysis and lipid synthesis might be reduced in the liver of the HE- group.

### Weighted gene co-expression analysis (WGCNA) and enrichment pathways of modules correlated to feed efficiency traits

**Figure 4:**
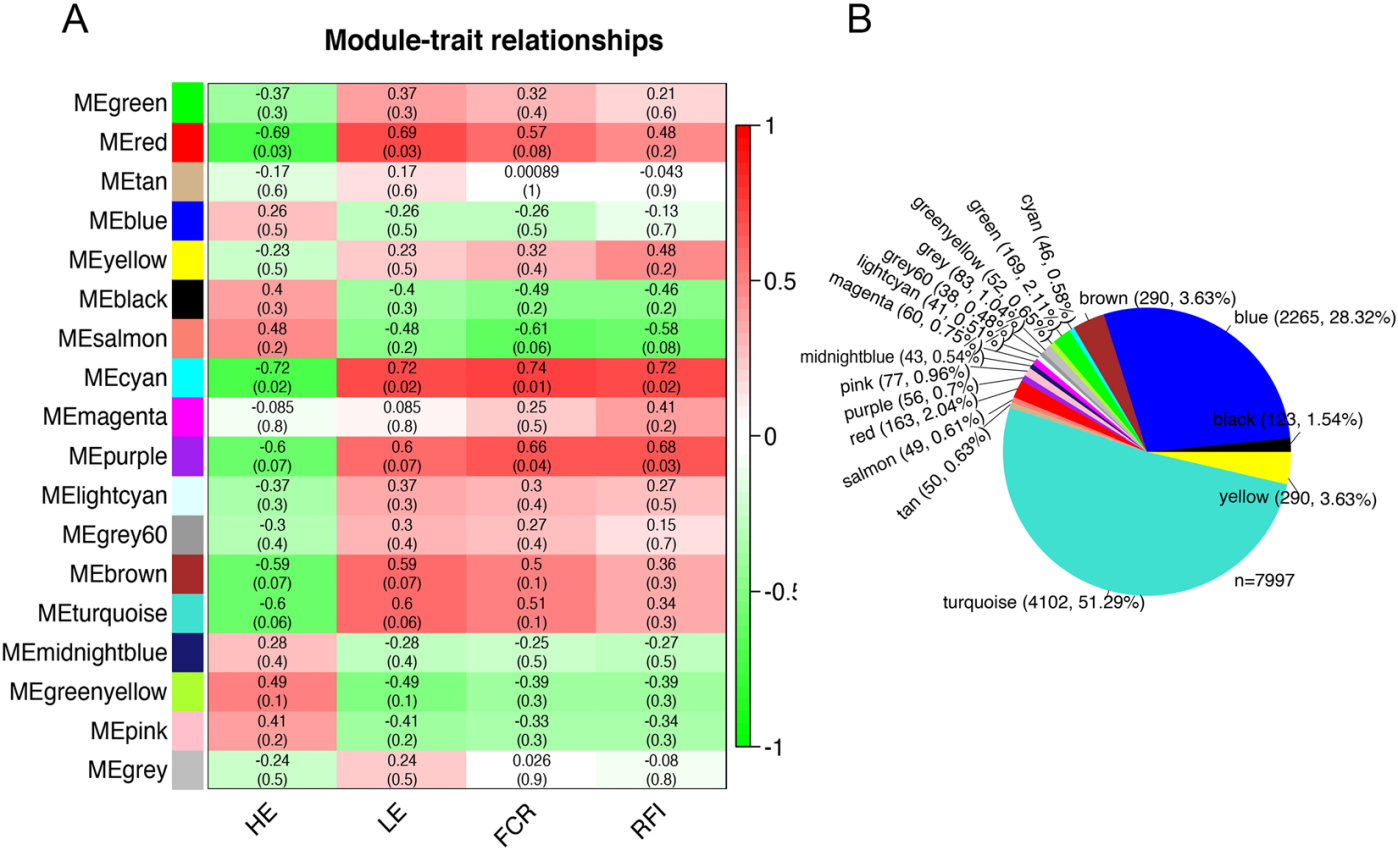
Weighted gene co-expression analysis (WGCNA). (**A**) Correlations between hepatic genes co-expression modules and feed efficiency traits of DLY pig. Modules represent the network of co-expressed genes and are named by different colors. Correlations are presented in the rectangles and the value in parentheses represent the *p*-value. (**B**) The number and percentage of genes in various modules.

WGCNA was conducted to identify gene co-expression modules that are correlated with the trait of interest (HE, LE, RFI, and FCR). A total of 18 co-expressed gene modules were identified and named by different colors (Fig. 4A). The list of genes in these modules was presented in Table S6. Among them, two-thirds (12/18) are negatively correlated and one-third (6/18) are positively correlated with HE- group. This may imply that high feed efficiency may be associated with a lower level of certain biological processes. Two of these modules were significantly positively associated with RFI and FCR, namely the MEcyan module (r = 0.72, p = 0.02; r = 0.74, p = 0.01) and the MEpurple module (r = 0.68, p = 0.03; r = 0.66, p = 0.04). The MEcyan module (r = −0.72, p = 0.02) and MEred module (r = −0.69, p = 0.03) were negatively correlated with HE- group, while positively correlated with LE- group. The MEcyan module clustered 46 genes, and 56 genes were clustered in MEpurple module, while 163 in MEred (Fig. 4B).

The functional enrichment analysis for the three modules significantly correlated with FE-related traits was conducted in KEGG and Reactome database and were presented in Table S7. The MEcyan and MEpurple module identified 20 significantly enriched pathways in KEGG database, while 4 in Reactome database (p.adjust <0.05) (Table 3). However, there was no significantly enriched pathway in MEred. Moreover, none of the significantly enriched terms were identified in GO analysis in the MEcyan, MEpurple and MEred modules (Table S8). Oxidative phosphorylation and thermogenesis were the most significantly enriched pathways identified in KEGG database, which contained 12 genes and related to energy consumption and negatively correlated with HE- group (Table 3). Three of four significantly enriched pathways enriched in Reactome database were correlated with energy metabolism, including “The citric acid (TCA) cycle and respiratory electron transport”, “Respiratory electron transport”, and “Respiratory electron transport, ATP synthesis by chemiosmotic coupling, and heat production by uncoupling proteins” (Table 3).

**Table 3:** A total of 20 significantly enriched pathways in KEGG database and 4 in Reactome database for MEcyan and MEpurple modules.

Pathway	*q*-value	Gene Names
**Reactome**		
The citric acid (TCA) cycle and respiratory electron transport	0.020278616	*NDUFV3, ADHFE1, NDUFB7, NDUFA11, NDUFA2*
Complex I biogenesis	0.020278616	*NDUFV3, NDUFB7, NDUFA11, NDUFA2*
Respiratory electron transport	0.020278616	*NDUFV3, NDUFB7, NDUFA11, NDUFA2*
Respiratory electron transport, ATP synthesis by chemiosmotic coupling, and heat production by uncoupling proteins.	0.042865302	*NDUFV3, NDUFB7, NDUFA11, NDUFA2*
**KEGG pathway**		
Oxidative phosphorylation	1.33E-09	*NDUFV3, NDUFA13, UQCR10, NDUFB7, LOC100525869, LOC100516480, COX5B, COX17, NDUFA11, COX7A1, NDUFA2, ATP6V0C*
Thermogenesis	3.18E-07	*NDUFV3, LOC100516527, NDUFA13, UQCR10, NDUFB7, LOC100525869, LOC100516480, COX5B, COX17, NDUFA11, COX7A1, NDUFA2*
Parkinson disease	3.18E-07	*NDUFV3, NDUFA13, UQCR10, NDUFB7, LOC100525869, LOC100516480, COX5B, NDUFA11, COX7A1, NDUFA2*
Non-alcoholic fatty liver disease (NAFLD)	3.72E-07	*NDUFV3, NDUFA13, UQCR10, NDUFB7, LOC100525869, LOC100516480, COX5B, NDUFA11, COX7A1, NDUFA2*
Huntington disease	3.72E-07	*NDUFV3, NDUFA13, UQCR10, NDUFB7, LOC100525869, LOC100516480, COX5B, NDUFA11, COX7A1, NDUFA2, POLR2J*
Alzheimer disease	7.96E-07	*NDUFV3, NDUFA13, UQCR10, NDUFB7, LOC100525869, LOC100516480, COX5B, NDUFA11, COX7A1, NDUFA2*
Cardiac muscle contraction	0.001847933	*UQCR10, LOC100525869, LOC100516480, COX5B, COX7A1*
Retrograde endocannabinoid signaling	0.002439495	*NDUFV3, NDUFA13, NDUFB7, GNB2, NDUFA11, NDUFA2*
Pentose and glucuronate interconversions	0.002655063	*GUSB, LOC100516628, UGT2B31*
Porphyrin and chlorophyll metabolism	0.007363959	*GUSB, LOC100516628, UGT2B31*
Sulfur metabolism	0.011099108	*SELENBP1, TST*
Drug metabolism - cytochrome P450	0.018779781	*ADH4, LOC100516628, UGT2B31*
Metabolism of xenobiotics by cytochrome P450	0.019524461	*ADH4, LOC100516628, UGT2B31*
Retinol metabolism	0.025126094	*ADH4, LOC100516628, UGT2B31*
Fatty acid metabolism	0.025126094	*FADS1, FADS2, CBR4*
Ascorbate and aldarate metabolism	0.025126094	*LOC100516628, UGT2B31*
Drug metabolism - other enzymes	0.025213695	*GUSB, LOC100516628, UGT2B31*
Chemical carcinogenesis	0.026132735	*ADH4, LOC100516628, UGT2B31*
Aminoacyl-tRNA biosynthesis	0.030796382	*VARS1, HARS1, MARS1*
Ribosome	0.033382135	*RPL26L1, MRPL14, RPS21, RPL31*

Significantly enriched pathways (*q*-value < 0.05) are listed with the pathway, *q*-value and gene names.

### Quantitative real-time PCR validation of six randomly selected DEGs

**Figure 5:**
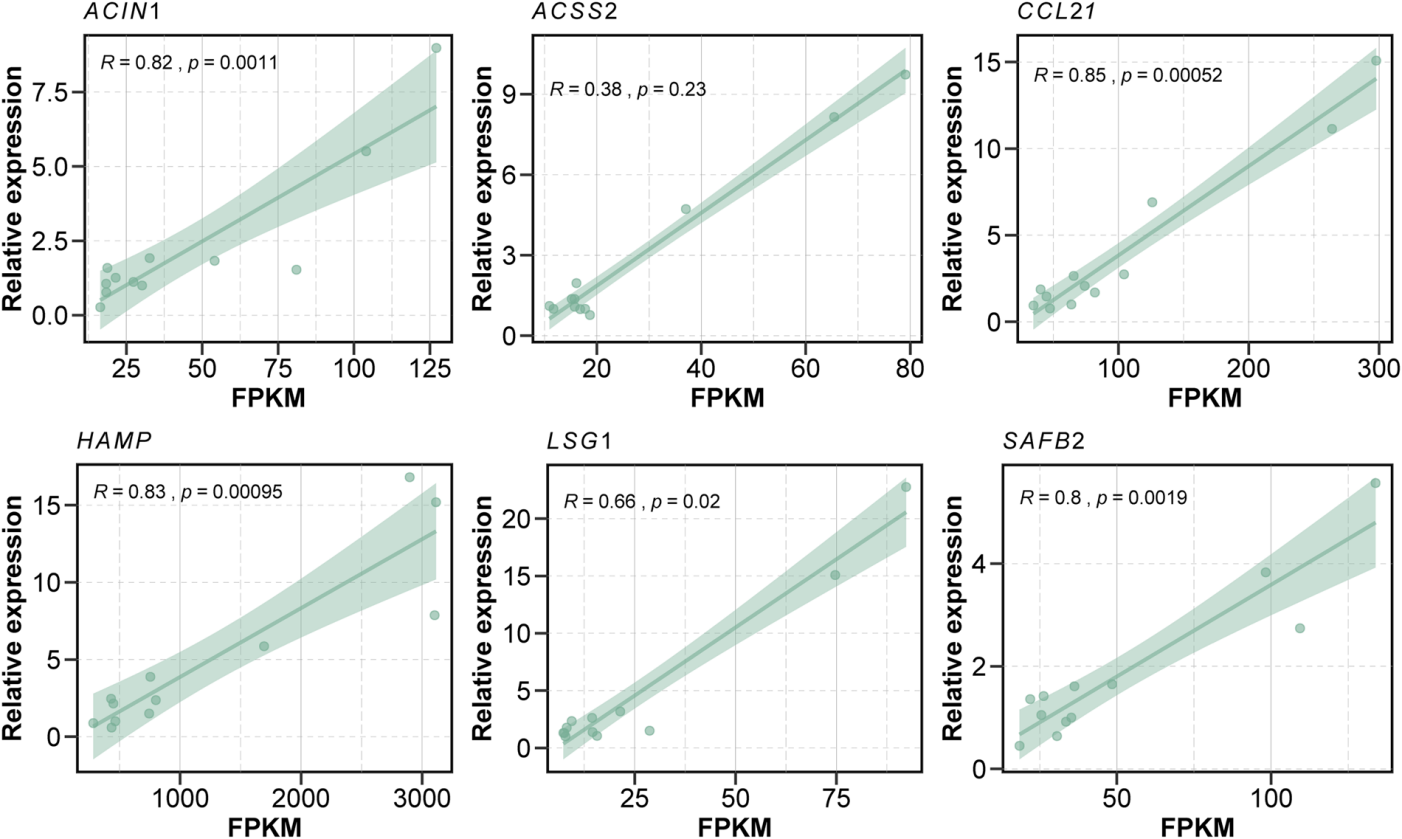
Correlation analysis of RNA-seq and quantitative polymerase chain reaction (qPCR) of 6 randomly selected DEGs. Six randomly selected DEGs were analyzed by real-time qPCR. The x-axis represents the fragments per kilobase of transcript per million mapped reads (FPKM) of each gene calculated by RNA-seq analysis, and the y-axis represents the relative expression of each gene calculated by qPCR. The correlation coefficient was calculated between the two measures.

The reliability of the DEGs identified by RNA-seq was validated by qPCR. Six DEGs (*ACIN1*, *ACSS2*, *CCL21*, *HAMP*, *LSG1*, and *SAFB2*) were randomly selected for qPCR. Moreover, all of 12 individuals from the HE- and LE- groups were selected for qPCR. A significant correlation (*P-value* < 0.05) between the gene expression data calculated by RNA-seq and the gene expression data calculated by qPCR was found in 5 selected DEGs (*ACIN1*, *CCL21*, *HAMP*, *LSG1*, and *SAFB2*) (Fig. 5). Although the *P-value* of *ACSS2* was more than 0.05, it had a trend line similar to the other 5 selected DEGs. These results revealed a significant correlation between the two measures and confirmed the reliability of the gene expression data identified by RNA-seq.

## Discussion

In this study, the DEGs, at the mRNA level, in the liver of the HE- and LE- groups were identified. Furthermore, the relevant pathways and candidate genes affecting the different FEs between the two groups were explored, illuminating the metabolic pathways and molecular mechanisms associated with the divergence in FE. We found that decreased glycolytic, energy consumption and lipid synthesizing potential in the liver may be associated with improved feed efficiency in DLY pigs.

The PPI analysis revealed some candidate genes associated with glycolysis (including *GCK*, *ENO2*, *HK3*, *PFKFB1*, and *PFKFB4*). Combining the results of GO and pathway enrichment analysis, *HK3*, *PFKFB1*, *GCK*, and *PFKFB4* were enriched in the most significantly enriched GO terms and pathways, all of which were downregulated in the HE- group. The *GCK* gene is involved in glycolysis and encodes hexokinase 4 that catalyzes glucose transfer to glucose-6-phosphate^25,26^. The overexpression of *GCK* increased glucose uptake and promoted glucose utilization in the liver^27^. Moreover, hepatic *GCK* mRNA expression was positively associated with the mRNA expression of lipogenic enzymes (*ACACB* and *FASN*) and de novo lipogenesis in the liver^26^. Thus, the hepatic glycolytic process probably stimulated hepatic lipid synthesis. The *ENO2* gene increases glucose uptake in the liver and participates in hepatic glycolysis by converting 2-phosphoglycerate into phosphoenolpyruvate^28^. The *HK3* (hexokinase 3) gene, one of the four hexokinase family members, is a catalytic enzyme in glycolysis^29^. Similarly, the *PFKFB1* and *PFKFB4* genes encode key enzymes involved in glycolysis^30,31^. In a previous study, Reyer found three adjacent SNPs in SSC13, two of which were located beside and in the *PFKFB4* gene in pigs, and these SNPs were significantly correlated with RFI^32^, suggesting that *PFKFB4* might be a candidate gene affecting FE. Liver glycolysis is one of the most important metabolic pathways regulating FE and is decreased in high-FE beef cattle^33^. Moreover, a study showed that genes involved in glycolysis were downregulated in high-FE pigs^34^.

Carbohydrate phosphorylation is the first step in glycolysis, in which glucose is phosphorylated to glucose-6-phosphate (G6P) by hexokinase^35^. In the fed state, G6P is metabolized to generate pyruvate through glycolysis. Pyruvate is the main glycolytic product and links glycolysis to lipogenesis. Pyruvate can be completely oxidized in mitochondria to generate ATP through the citric acid cycle and oxidative phosphorylation^25^. Glycolysis produces ATP to provide energy for growth, which is an extremely inefficient way of producing energy. In the complete oxidation of pyruvate, approximately 40% of the energy produced is used to synthesize ATP, while the remaining energy (approximately 60%) is lost as heat energy^36^. Combining with the results of WGCNA, oxidative phosphorylation, thermogenesis, and energy metabolism-related pathways (including TCA cycle, respiratory electron transport, ATP synthesis, and heat production) were significantly enriched in MEcyan and MEpurple modules, and all of these biological processes occurred in mitochondria and are related to energy consumption^25,37^. The MEcyan and MEpurple modules were negatively related to HE- group, indicating that higher efficiency in HE- group might due to lower energy consumption in the liver. These results implied that decreased rates of hepatic glucose metabolism, oxidative phosphorylation, thermogenesis, and energy metabolism might result in fewer energy losses in the HE- group. Hence, we hypothesized that the HE- group had more efficient energy utilization and a higher FE because the decreased glycolysis process and reduced energy losses. Correspondingly, previous analyses of FE revealed that the genes associated with the glycolytic pathway and mitochondrial activity were downregulated in the liver and muscle tissues of high-FE pigs. Less energy was lost due to the decreased glycolytic potential and mitochondrial activity in the liver, which might result in higher energy efficiency in HE- group^19,34,38–40^.

Furthermore, pyruvate can provide a carbon source for lipogenesis in the liver. The conversion of glucose to lipids in the liver results in an approximately 23% energy loss^41^, which is much higher than the amount of energy lost by protein deposition in the muscle^42^. Previous studies focusing on muscle tissue revealed that high-FE pigs accumulated more muscle mass compared with low-FE pigs^42,43^, which indicated that high-FE pigs needed to consume more glucose in the muscle to generate ATP for protein deposition. In our study, the HE- group had lower glucose metabolic and lipid synthesizing potential in the liver, so we speculate that more glucose is consumed in the muscle tissue to provide ATP for protein deposition in the HE- group. Protein synthesis has a higher energy efficiency than lipid synthesis; thus, the HE- group exhibited higher feed efficiency. Our findings are consistent with previous studies and support the assumption that the HE- group had less heat production and greater energy utilization related to decreased glycolytic processes in the liver, which may have positive effects on FE in commercial DLY pigs.

The liver is the main organ that synthesizes fatty acids and other lipids. The precursor substances for the synthesis of fatty acids are mainly derived from glucose metabolism, especially glycolysis^25^. In our study, lipid synthesis-related pathways were significantly enriched in the pathway enrichment analysis (among the top 10 significantly enriched pathways, 4 pathways were related to lipid synthesis), and genes involved in lipid synthesis, including *ACACB*, *CYP21A2*, *CHKA*, *SCD*, were downregulated in the HE- group. The *ACACB* gene is a known candidate gene for lipid metabolism and is related to the *de novo* synthesis of fatty acids and other lipids^44^. Both *CYP21A2* and *CHKA* are involved in the synthesis of cholesterol, steroids and other lipids and are candidate genes that affect lipid synthesis in pigs^45,46^. Lipid synthesis in the liver was negatively related to the FE trait in pigs, which has been reported in previous studies^47,48^. Several studies found that genes involved in lipid metabolisms, such as fatty acid, steroid, and cholesterol biosynthesis, were downregulated in the liver of low-RFI (more efficient) pig^16,49,50^ and cattle^51^.

The PPI analysis indicated that the genes associated with lipid synthesis (including *ACACB*, *SCD*, *FASN, HSD17B1*, and *CYP21A2*) were candidate genes that affected the FE in commercial DLY pigs. The *SCD* gene, which encodes stearoyl CoA desaturase and promotes the synthesis of fatty acid in pigs^52^, is a candidate gene that correlates with FE^14^. Previous studies revealed that the *SCD* gene was downregulated in more efficient pigs^52–54^, and the high-FE pigs had a reduction of lipid synthesis and accumulation. The *FASN* gene is a fatty acid synthase that plays an important role in the *de novo* synthesis of fatty acids^55^ and was downregulated in high-FE pigs^52–54^. In the current study, the *SCD* and *FASN* genes were downregulated in the HE- group compared with the LE- group. Moreover, by analyzing the top 5 significantly upregulated DEGs, we found that *DLK1* was related to lipid metabolism, and *DLK1* was upregulated by a log_2_ (Foldchange) of 3.81 in the HE- group. Previous studies indicated that the overexpression of *DLK1* would suppress lipid synthesis, and this gene was upregulated in more efficient pigs and cattle^51,56^. Consistent with previous studies, our results indicated that the increased FE in the HE- group might be associated with the reduction of lipid synthesis and accumulation in the liver.

## Conclusion

In this study, we investigated the liver transcriptome of 6 extremely high feed efficiency pigs (HE- group) and 6 extremely low feed efficiency pigs (LE- group). A total of 507 DEGs were found between the HE- and LE- groups. GO and pathway enrichment analyses revealed that the DEGs were mainly enriched in glycolysis and lipid synthesis. The vast majority of DEGs involved in glycolysis and lipid synthesis were downregulated in the HE- group, such as *SCD*, *ACACB*, *FASN*, *GCK*, and *ENO2*. The expression patterns of these genes suggest that the related pathways might influence feed efficiency in pigs. Moreover, the results of WGCNA indicated that oxidative phosphorylation, thermogenesis, and energy metabolism-related pathways were decreased in HE- group, which resulted in higher energy efficiency in it. Briefly, the results indicated that the HE- group may have decreased glycolytic, energy consumption and lipid synthesizing potential in the liver, thereby increasing energy efficiency. Our findings provide an understanding of the molecular mechanisms in the liver in regulating the feed efficiency of DLY pigs. The key pathway and candidate genes identified in this study are potentially useful for improving porcine feed efficiency.

## Methods

### Ethics statement

The experimental procedures used in this study met the guidelines of the Animal Care and Use Committee of the South China Agricultural University (SCAU) (Guangzhou, People’s Republic of China), and every effort was taken to minimize animal suffering. All animal experiments in this study were approved by the Animal Care and Use Committee (ACUC) of the SCAU (approval number SCAU#0030).

### Animals and tissues

In this study, a total of 225 commercial Duroc × (Landrace × Yorkshire) sows, provided by Guangdong Wen’s Foodstuffs Group Co., Ltd., (Yun fu, China), were selected as the experimental animals. During the experiment, the pigs were housed in an environment-controlled shed, and feed and water were offered ad libitum. The phenotypic data of 225 sows were recorded by the Osborne FIRE pig performance test system (Osborne, KS, USA). The recording period was approximately 12 weeks, during which time the weight of the animals was measured from approximately 30 kg of body weight (BW) to 100 kg BW. Each individual had a unique electronic identification tag on its ear that could be captured by an automatic feeder. Each individual’s feeding time, feeding duration, feed consumption, and body weight were recorded at each visit to the feeder. The standard A-scan and contact B-scan ultrasonography were used to measure back fat (BF) of pigs in approximately 100 kg BW. The FCR and RFI were calculated for each individual during the trial. The RFI calculation method was similar to that of Cai *et al*.^57^. The RFI was estimated by the linear regression of DFI on metabolic BW at mid-test (MWT), average daily gain from 30 to 100 kg (ADG), and BF. MWT was equal to [(BW at on-test + BW at off-test)/2] ^0.75^. Then, the RFI values of all individuals were ranked, and 6 pigs with extremely high-FE and 6 pigs with extremely low-FE were selected and designated as the HE- group and LE- group, respectively. Finally, a correlation analysis was conducted to calculate the correlation between RFI and FCR to verify the selection of 12 individuals.

At the end of the experiment, all 12 individuals from the HE- and LE- groups were slaughtered, and liver tissues (the middle portion of the left lateral lobe) were collected immediately. These samples were rapidly frozen in liquid nitrogen and stored at −80 °C.

### RNA extraction and sequencing

Total RNA was extracted from all 12 liver tissue samples using Total Kit II (OMEGA, USA) and the procedures and standards were performed according to the manual. The quantification and quality of RNA were assessed by a NanoDrop2000 microspectrophotometer (Thermo Scientific, Wilmington, DE, USA). The concentration of the mRNA ranged from 624 to 1218 ng/μl, and the absorbance (A260/280) of all samples was between 1.8 and 2.1. Besides, an Agilent 2100 Bioanalyzer device (Agilent Technologies, Santa Clara, CA, USA) was used to assess the integrity of the RNA. The RNA integrity value (RIN) of our samples ranged between 6.2–8.8. The cDNA library was constructed using a TruSeq RNA Library Prep Kit v2 (Illumina, San Diego, CA, USA) according to the manufacturer’s instructions, where mRNA was purified and enriched from 1 μg of each of the total RNA samples and then fragmented. After quality control, the libraries were sequenced on an Illumina HiSeq 4000 platform.

### Read alignment and differential expression analysis

The raw reads of each sample were discriminated based on the indexing adaptors. The FastQC^58^ software was used to evaluate the quality of the reads. Then, the adapter sequences and low-quality reads (the reads that adapter contamination is greater than 5 bp, Q20 ratio does not reach 85% or containing N ratios greater than 5%) were filtered out, and the high-quality reads were available for downstream analysis. The STAR: ultrafast universal RNA-seq aligner STAR_2.3.0^59^ was used to map the sequencing reads with the reference genome (*Sus scrofa* 11.1), and all the options were set as STAR default values. The HTSeq^60^ software was used to generate the read count tables for further differential expression analysis.

Differential expression analysis was performed between the HE- and LE- groups by using the Gfold (V1.1.2)^61^ software and the methods described by Audic and Claverie^62^. The Gfold (V1.1.2) software was used to count the expression level of the reads and convert them into FPKM (Fragment Per Kilobase of exon model Per Million mapped reads). The expression fold change between the two groups was calculated by the methods described by Audic and Claverie, and the Benjamini-Hochberg (BH) method was performed to calculate *q*-value for multiple testing. All genes were filtered by the criteria of |log_2_(Foldchange)| > 1 and *q*-value < 0.001. Genes with log_2_(Foldchange) > 1 and *q*-value < 0.001 were defined as upregulated DEGs, while the gene with log_2_(Foldchange) < −1 and *q*-value < 0.001 were defined as downregulated DEGs.

### GO and pathway enrichment analysis of DEGs

To explore the major metabolic pathways and cell signaling pathways related to FE, Gene Ontology (GO) and pathway enrichment analysis were carried out by KOBAS 3.0 (http://kobas.cbi.pku.edu.cn/anno_iden.php)^63^, and both the Reactome and Kyoto Encyclopedia of Genes and Genomes (KEGG) database were used for pathway enrichment analysis. The BH method was applied to adjust the *P-value* for multiple testing. The GO terms or pathways meeting the screening criteria (with *q*-value <0.05) were the significantly enriched terms or pathways.

### Protein-protein interaction network construction

A protein-protein interaction (PPI) analysis of DEGs was implemented in the Search Tool for the Retrieval of Interacting Genes (STRING) database. The intensity of interaction among the input genes was evaluated and the hub gene was determined according to the degree of relationship to other genes. Then, the interaction network diagram of these genes was plotted by the Cytoscape (3.6) software^64^.

### Weighted gene co-expression analysis (WGCNA)

Gene co-expression network analysis was performed using the R package WGCNA^65^. Detailed steps are as follows. (1) Data input and cleaning: The gene expression matrix (genes that expression with variance variation accounting for top 30%) and the phenotypic matrix (including HE, LE, RFI, and FCR) for 12 individuals were used for subsequent analysis. In the phenotypic matrix, the RFI and FCR values in the matrix came from the RFI and FCR original values of each of 12 individuals. Six individuals belonging to high-FE group were marked as 1 and the remaining 6 as 0 for the “HE” values of the phenotypic matrix. Similarly, six individuals belonging to low-FE group were marked as 1 and the remaining 6 as 0 for the “LE” values of the phenotypic matrix. Gene and individual quality control were performed with the default settings of the R package WGCNA. (2) Best soft-Threshold Confirmation: Using the pickSoftThreshold function of the R package WGCNA to analyze the expression matrix obtained in the first step, the most appropriate soft threshold value was 7. (3) Network construction and module detection: Using the blockwiseModules function to analyze the expression matrix obtained in the first step for module detection with power = 7 and mergeCutHeight = 0.2, the gene set was divided into 18 modules. (4) Relating modules to phenotypic traits and identifying important genes: Correlation analysis and significance test were performed on the phenotypic matrix obtained in the first step and the modules obtained in the third step. Modules with statistically significant (*p*-value <0.05) correlations were selected for downstream analysis. (5) Functional annotation of significant module genes: gene pathway analysis and gene function annotation were analyzed using R package clusterProfiler^66^ with default parameters. Biological terms were considered significant if the adjusted *p*-value was less than 0.05.

### Real-time quantitative PCR

To validate the results of the differential expression analysis from the RNA-seq data, the relative expression levels of 6 randomly selected DEGs were detected by real-time quantitative polymerase chain reaction (qPCR) technology. A total of 6 HE- and 6 LE- pigs were selected for qPCR. RNA samples were prepared by the methods mentioned above. Reverse transcription was performed by the PrimeScript^TM^ RT reagent kit (Takara, Japan). Then, all qPCR reactions were performed in a QuantStudioTM 7 flex device (Invitrogen Life Technologies, Carlsbad, CA, USA) following the manufacturer’s instructions and three biological replicates were used in the experiment. The parameters used in the qPCR reaction were: denatured at 95 °C for 5 min; performed 40 PCR cycles (95 °C, 10 s; 60 °C, 15 s; 72 °C, 20 s); dissolution curve (95 °C, 15 s, 55 °C, 15 s, 95 °C, 15 s). Thereafter, the comparative Ct method^67^ was performed to quantify the gene expression of 6 selected genes in 12 individuals. The primer sequences of these genes were designed by the Oligo 7.0 software, and the details of the primers are displayed in Table S9.

## Supplementary information

Supplementary Tables.

## Data Availability

The raw reads have been submitted to the NCBI Sequence Read Archive database (SRA) under BioProject accession number of PRJNA578377 and SRA accession number SRR10315359 - SRR10315370.
